# Molecular dissection of the human antibody response to the structural repeat epitope of *Plasmodium falciparum *sporozoite from a protected donor

**DOI:** 10.1186/1475-2875-3-28

**Published:** 2004-07-29

**Authors:** Jonathan A Chappel, William O Rogers, Stephen L Hoffman, Angray S Kang

**Affiliations:** 1 Department of Molecular Biology, The Scripps Research Institute, 10550 North Torrey Pines Road, La Jolla, CA 92037, USA; 2 Malaria Program, Naval Medical Research Center, Silver Spring, MD 20910-7500, USA; 3 Present address: Naval Medical Research Unit #3, Ghana Det, c/o Department of State, 2020 Accra Place, Washington, DC 20521-2020, USA; 4 Present address: Sanaria Inc, 12115 Parklawn Drive Suite L, Rockville, MD 20852, USA; 5 Present address: Avanir Pharmaceuticals Inc, 11388 Sorrento Valley Road, San Diego, CA 92121, USA

## Abstract

**Background:**

The circumsporozoite surface protein is the primary target of human antibodies against *Plasmodium falciparum *sporozoites, these antibodies are predominantly directed to the major repetitive epitope (Asn-Pro-Asn-Ala)_n_, (NPNA)_n_. In individuals immunized by the bites of irradiated *Anopheles *mosquitoes carrying *P. falciparum *sporozoites in their salivary glands, the anti-repeat response dominates and is thought by many to play a role in protective immunity.

**Methods:**

The antibody repertoire from a protected individual immunized by the bites of irradiated *P. falciparum *infected *Anopheles stephensi *was recapitulated in a phage display library. Following affinity based selection against (NPNA)_3 _antibody fragments that recognized the PfCSP repeat epitope were rescued.

**Results:**

Analysis of selected antibody fragments implied the response was restricted to a single antibody fragment consisting of V_H_3 and V_κ_I families for heavy and light chain respectively with moderate affinity for the ligand.

**Conclusion:**

The dissection of the protective antibody response against the repeat epitope revealed that the response was apparently restricted to a single V_H_/V_L _pairing (PfNPNA-1). The affinity for the ligand was in the μM range. If anti-repeat antibodies are involved in the protective immunity elicited by exposure to radiation attenuated *P. falciparum *sporozoites, then high circulating levels of antibodies against the repeat region may be more important than intrinsic high affinity for protection. The ability to attain and sustain high levels of anti-(NPNA)_n _will be one of the key determinants of efficacy for a vaccine that relies upon anti-PfCSP repeat antibodies as the primary mechanism of protective immunity against *P. falciparum.*

## Background

Malaria threatens public health in regions of the world where more than a third of the human population lives [1,2]. It has been shown that immunization with radiation-attenuated *Plasmodium *sporozoites, the infective stage of the malaria parasite, confers protective immunity [3,4]. The role of specific antibody in conferring protection was demonstrated with passive administration of murine mAbs directed against the major repeat epitope of the circumsporozoite (CS) protein [5] in a rodent model. The corresponding epitope of the human malaria parasite *Plasmodium falciparum *is contained within the repeat tetramer peptide (Asn-Pro-Asn-Ala)_n_, (NPNA)_n _[6]. In some studies of volunteers protected against malaria by immunization with radiation attenuated *P. falciparum *sporozoites, protected individuals had significant elevations of anti-repeat antibodies (>19 μg/ml) [7].

With the advent of recombinant combinatorial antibody technology [8,9] and phage display [10-13] it is possible to attempt to dissect the human antibody response against a wide range of pathogens. In order to further investigate the role of the human antibody response in *P. falciparum *sporozoite induced protection, a phage display library of antibody gene fragments isolated from the peripheral blood lymphocytes of such a protected donor (WR5) [7] was assembled. Recombinant antibodies against the PfCSP structural repeat (NPNA)_3 _epitope were selected. Recognition was restricted to a single antibody designated PfNPNA-1, encoded by V_H_3 and V_κ_I families. This restricted humoral response has implications for rational vaccine design and the potential use of this human monoclonal antibody to prevent *P. falciparum *infection.

## Methods

### RT-PCR of Immunoglobulin genes

A human volunteer (WR5), who was previously exposed to the bites of γ-irradiated *P. falciparum *infected *Anopheles *mosquito's and subsequently shown to be protected against a non-irradiated parasite challenge, donated lymphocytes by leukophoresis five days after a booster challenge (appropriate informed consent was obtained) for details see Egan et al., [7]. The irradiated sporozoite immunization protocol was approved by the Naval Medical Research Institute's Committee for the Protection of Human Subjects in accordance with the US Navy regulation (SECNAVINST3900.39B) governing the use of human participants in medical research. Total RNA was extracted from 2 ml of packed cells using an RNA isolation kit (Stratagene, La Jolla, CA) with a modified protocol [9]. The equivalent of 2.5 μg total RNA template were used in each cDNA synthesis reaction using reverse transcriptase (Invitrogen, CA) with oligonucleotide oligo dT or 3 'HuVH (5'GCCCCCAGAGGTGCTCTTGGA-3', anneals in CH1 domain) following the instructions provided by the supplier.

The genes encoding variable heavy (V_H_) and the kappa chain (κ) were accessed by RT-PCR and combined by overlap extension PCR, resulting in shuffling of the V_H _and the V_L _domains. The V_H _PCR amplification was carried out with the cDNA template generated using the 3'HuVH primer. The V_H _domains were amplified using 5'HuVHA and 3'HuVH-Link 3' designed to anneal with the sequence corresponding to the first β-strand of the CH1 domain and overlap with the 5'HuVk primer. The κ chains were amplified using 5'HuVk and the 3'Hukappa primers. The V_H _and the κ chain PCR products were combined by overlap extension PCR using a V_H _flanking primer 5'HuVHB (to introduce a *NheI *site) and the 3'HuKappa primer.

### Oligonucleotide primer sequences

#### 5'HuVk

5'-TATTAGCGGCCGCCCAACCAGCCATGGCCGAEFIJLOPETGACBCAGTCTCC-3' (where B=G+C+T, S=G+C, E = 50%A+33%C+17%T, F = 83%A = 17%G, I = 83%T+17%C, J = 50%T+33%C+17%G, L = 67%G+17%T+17%C, O = 67%T+17%A+17%C, and P = 83%G+17%C)

#### 3'HuKappa

5'-TCCTGAAGCTTGACGACCTTCGATCTCTCCCCTGTTGAAGCTCTT-3'

#### 5'HuVHA

5'-SAGGTGCAGCTGSTGSAGTCTGG-3'

#### 5'HuVHlink3'

5'-GGCTGGTTGGGCGGCCGCTAATATGGAGGAGGGTGCCAGGGGGAAGAC-3'

#### 3'HuVHB

5'-GTTTCGCTAGCGTAGCTCAGGCTSAGGTGCAGCTGSTGSAGTCTGG-3' 

**Figure 1:**
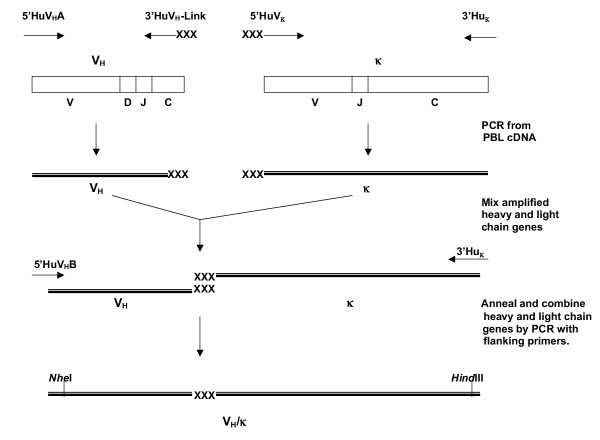
**V_H_/κ library construction. **A schematic diagram of the steps involved in constructing a V_H_/κ library from mRNA isolated from PBL.

The procedural steps are illustrated in Figure 1.

### Cloning PCR fragments into pORFES and JC-M13-88

**Figure 2:**
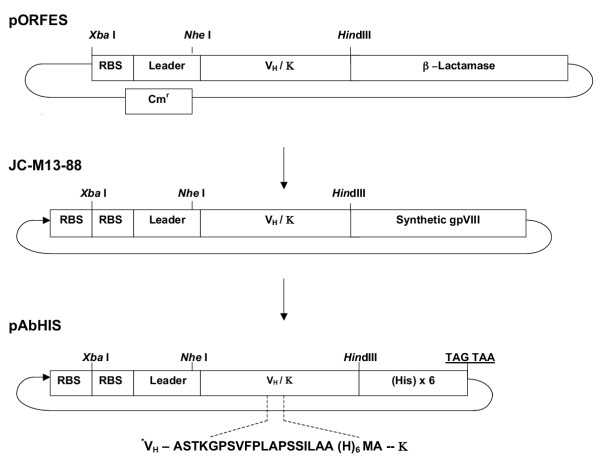
Illustration of vectors pORFES, JC-M13-88 and pAbHIS.

The PCR amplified V_H_/κ products were digested with restriction enzymes *NheI *and *HindIII, *and ligated into pORFES [14]. An aliquot of *E. coli *transformed with the ligation mixture was plated with and without carbenicillin selection, to determine the number of functional inserts. The V_H_/κ coding sequences are directionally inserted for expression between an OmpA leader peptide (to direct the polypeptide into the periplasm), and the β-lactamase. Functional full-length V_H_/κ β-lactamase fusion polypeptide is secreted into the periplasm. Bacteria harbouring plasmids conferring antibiotic resistance may be positively selected. The V_H_/κ coding insert may be readily transferred as a *XbaI*-*HindIII *fragment into the JC-M13-88 phage vector to display the insert polypeptide as a gpVIII fusion. The selected "functional" library of V_H_/κ inserts were excised from pORFES using *XbaI *and *HindIII*, ligated into pre-digested JC-M13-88 [4], and transformed into *E. coli *(XL1-Blue: Stratagene). Phage was produced overnight at 37°C in the presence of 1 mM IPTG, unless otherwise stated. A schematic outline of the vectors is shown in Figure 2.

### Phage panning

The peptide (NPNA)_3_C (Chiron Mimotopes Peptide Systems, San Diego, CA.) was conjugated to BSA using Imject Activated Immunogen kit (Pierce, Rockford, IL) according to the manufacturers guidelines. ELISA plates (Dynatech Immunlon I, Alexandria, VA) were coated with BSA or (NPNA)_3_C-BSA and used in phage panning experiments essentially as described elsewhere [5]. To blocked antigen coated wells a total of 4 × 10^10 ^plaque forming units (pfu) of the phage library in dilution buffer (PBS pH 7.2, Tween-20 0.05%, BSA 0.1%, NaN_3 _0.02%) was added (1 × 10^10 ^plaque forming units (pfu) per well). After 4 h the wells were washed and the bound phage were eluted by applying either 0.1 M glycine-HCl, pH2.2 or a solution of the free peptide (NPNA)_3 _(~8 μM) dissolved in dilution buffer, for 15 min at ambient temperature. An aliquot of the phage elute was titered, and the remainder was used to propagate phage for further rounds of panning. The three-domain single chain antibody retains the kappa constant domain thus permits plaques filter lifts to be probed with anti-human kappa chain antibodies for immunodetection. V_H _and V_L _coding sequences were determined by sequencing of replicative form (rf) phage DNA prepared from κ-positive plaques, using the oligonucleotides primers:

3'Seq VH-JC130 (5'-CGGCCATGGCTGGTTGGGCGGCC-3') and

3'Seq VL-JC128 (5'TTCAACTGCTCATCAGATGGCGG-3').

### Expression of PfNPNA-1 V_H_/*k *in *E. coli*

The expression vector pAbHIS, was constructed by modification of pUC18. The β-galactosidase coding region was removed and *XbaI*-*HindIII *sites introduced upstream of a sequence encoding a six histidine tail. Insertion of V_H_/κ coding sequence selected by phage display as *XbaI*-*HindIII *fragment would result in the expressed polypeptide being secreted into the periplasmic space with a hexa-histidine tag. The plasmid pAbHIS was constructed by PCR modification of pUC18 using the primers PUCSpe-JC127(5'-TCATCATACTAGTAACGACACCCGCCAACACCC-3') and M13-JC118 (5'-AAGCTTATGATGTCTAGAGCTGTTTCCTGTGTGAA-3'). A pair of annealed oligonucleotides designed to encode a 6×His tag were ligated into the *HindIII *digested plasmid to complete pAbHIS. The selected PfNPNA-1 V_H_/κ gene was excised from the rf JC-M13-88 DNA by digestion with *XbaI *and *HindIII *and ligated into similarly digested pAbHIS. An additional 6×His-coding pair of oligonucleotides was ligated into the PfNPNA-1 V_H_/κ linker sequence as NotI-NcoI insert. The expression of PfNPNA-1 V_H_/κ in *E. coli *D29A1 cells at 25°C, and the isolation of bacterial periplasmic material was performed as described [16] with modifications; Dnase I n(1 μg/ml) and MgCl_2 _(20 mM) were added, the bacterial suspension was incubated on ice for a further 20 min before final centrifugation step. The periplasmic extract was passed over Ni-NTA resin (Qiagen), washed and the PfNPNA-1 V_H_/κ was eluted with 300 mM imidazole. SDS PAGE and western blotting were used to asses purity and integrity of the expressed V_H_/κ polypeptide during the purification procedure (data not shown). Purified PfNPNA-1 V_H_/κ was quantified spectrophotometrically assuming an OD at 280 nm of 1 = 0.72 mg/ml protein.

### ELISA affinity and specificity determination

ELISA Plates (Dynatech Immunlon I) were coated with (NPNA)_3_C-BSA (10 μg/ml). Dilutions of the peptide (NPNA)_3 _were made in dimethyl formamide (DMF) before mixing with the PfNPNA-1 V_H_/κ diluted in PBST. Aliquots of 0.1 ml were added to duplicate wells, incubated for 2 h at 37°C. In all wells the final concentration of DMF was 1% (v/v). After washing 4 times with PBST, anti-human kappa chain alkaline phosphatase conjugate diluted 1:1000 in PBST was added and incubated as before. The wells were washed 4 × with PBST and rinsed 1× with PBS and substrate *p*-nitrophenyl phosphate was added, the absorbance was determined at 405 nm

The binding of immune serum (WR5), non-immune serum and PfNPNA-1 V_H_/κ to R32tet32, recombinant hepatitis core containing (NANP)_4 _peptide sequence and (NPNA)3C-BSA conjugate coated microtiter plate well was determined by ELISA essentially as described above. The serum(s) and the recombinant PfNPNA-1 V_H_/κ were diluted 1/16 and 1/10 respectively.

### Phage ELISA

Phage at 1 × 10^12 ^pfu/ml in dilution buffer were applied (0.1 ml/well) to duplicate wells coated with (NPNA)_3_-C-BSA or BSA (10 μg/ml). After incubation at ambient temperature for 4 h, plates were washed with PBST. The bound phage was detected with sheep anti-M13 antibodies (5'-prime 3'-prime), followed by rabbit anti-sheep alkaline phosphatase antibodies in PBST added sequentially for 1 h at 37°C. Plates were washed and developed as described above.

### Indirect immunofluorescence assay (IFA) on *P. falciparum *sporozoites

The PfNPNA-1 V_H_/κ was compared with a well-characterized murine monoclonal anti-*Pf *repeat antibody 2A10 [17,18] in IFA. All incubations were at 37°C in a humid container. Printed multiwell slides coated with *Plasmodium falciparum *NF54 strain sporozoites were either fixed in ice cold acetone for 10 min or used unfixed. Slides were first blocked with 4%BSA in PBS for 1 h. Antibodies diluted in PBST were applied for 2 h, then slides were washed 4× with PBS and fluoroscein-conjugated anti-human kappa chain or anti-mouse immunoglobulin (Sigma) was applied, diluted 1:25 in PBST. After 2 h slides were washed as above and mounted in SlowFade anti-fade reagent (Molecular Probes, Eugene, OR) and viewed by fluorescence microscopy.

### Other antibodies

The murine mAb 2A10 [17,18] (IgG2b, κ), which recognizes the (NANP)_3 _sequence of the *P. falciparum *CSP was provide as whole ascitic fluid (a kind gift from Dr P. Sinnis New York University). Concentration of the whole IgG was estimated using a standard antibody capture ELISA. Immune IgG (denoted (Vol-IgG) was purified from serum of the immune volunteer (WR5), donated at the time of lymphophoresis using Protein A Sepharose (Pharmacia) and quantified assuming OD at 280 nm of 1.0 represents 0.8 mg/ml IgG. Within the Vol-IgG, the proportion of (NPNA)_3 _specific IgG with κ or λ light chains were determined by ELISA (data not shown).

## Results

### Library construction

Sera from the protected individual (WR5) [7] contained antibodies against the PfCSP, which were predominantly IgG/κ and against the structural repeat peptide as determined by ELISA. Gene fragments encoding V_H_/κ single chain antibodies were amplified and assembled by PCR from cDNA derived from the peripheral blood lymphocytes of the immune donor WR5 (as outlined in Figure 1). The library of PCR amplified V_H_/κ sequences were inserted into pORFES [14] and an aliquot compared for number of functional inserts by selecting in the presence of either chloramphenicol (total transformation events) or chloramphenicol and carbenicillin (functional inserts). Approximately half of the initial library contained non-functional domains (data not shown). The remainder of the library was selected on 100 μg/ml carbenicillin, yielding a primary library of 1.3 × 10^6 ^members, these V_H_/κ sequences were transferred to the phage display vector JC-M13-88 [15] with ten fold over representation of the primary library.

### Panning

Samples of the V_H_/κ-phage library were subjected to four rounds of panning on (NPNA)_3_C-BSA coated wells. Both the acid and peptide elution strategies yielded significantly greater numbers of phage after four cycles of panning on (NPNA)_3_C-BSA when compared to panning on BSA alone (Table 1). Analysis of fifteen individual phage after the fourth round of panning on (NPNA)_3_C-BSA eluted with free peptide revealed, twelve kappa positive phage, of these three clones (NP 04, 12, 13) were positive in the phage ELISA for binding to (NPNA)_3_C-BSA and were encoded by an identical sequence, henceforth denoted PfNPNA-1. Prior to panning ten kappa positive clones were randomly selected for sequencing (R 01-10; Table 2). The PfNPNA-1 V_H _and V_L _sequences were members of the V_H_3 and V_κ_I families respectively and were not found amongst the random sampling of phage prior to panning. In an independent experiment with phage propagated at 30°C, but otherwise an identical panning procedure 12 out of 12 selected phage clones were identical to PfNPNA-1. Likewise, phage selected by acid elution and evaluated by ELISA for binding to (NPNA)_3_C-BSA were all identical to PfNPNA-1. Despite extensive sampling of phage that were positive in the phage ELISA for binding to (NPNA)_3_C-BSA (n = 25), only the PfNPNA-1 sequence was observed.

**Table 1:** Phage panning experiments ELISA plates (Dynatech Immulon I) were coated with BSA or (NPNA)_3_C-BSA and used in phage panning experiments. To the blocked antigen coated wells a total of 4 × 10^10 ^pfu of the phage library in dilution buffer were added 1 × l0^10 ^pfu per well. After 4 h the wells were washed and phage eluted by applying either 0.1 M glycine-HCl pH 2.2 or a solution of the free peptide (~8 μM) (NPNA)_3 _dissolved in dilution buffer for 15 min at ambient temperature. An aliquot of the phage eluate was titered and the output determined.

**Eluate after**	Coating antigen / Elution method (×l0^5 ^pfu)*			
**panning rounds**	**BSA / acid**	**BSA /(NPNA)_3_**	**(NPNA)_3_C BSA /acid**	**(NPNA)_3_C BSA /(NPNA)_3_**

1	2.9 (0.38)	0.82 (0.032)	3.0 (0.34)	0.51 (0.024)
2	1.4(0.03)	0.24 (0.020)	3.5 (0.24)	1.5 (0.028)
3	1.2(0.06)	0.47 (0.020)	4.3 (0.024)	12 (0.68)
4	13 (0.70)	1.0(0.032)	170 (30)	370 (20)

* Figures represent the mean of the total plaque forming units eluted by either acid or excess free peptide, after repeated panning against BSA or (NPNA)_3_C-BSA. Values for the standard deviation are shown in brackets ().

**Table 2:** V_H _and V_L _assignments and alignment of CDR 3 sequences The selected (NP 04, 12, 13 designated Pf NPNA-1 bind to the repeat epitope), all other NP clones were randomly picked after the panning procedure and were subsequently shown not to be reactive with the repeat epitope. Non-selected (R01-10) were randomly picked from the library prior to initiating panning. The peptide sequence of the heavy and light chain complementarity-determining region 3 (CDR3) is shown below. V_H_/V_L _families, segments and the number of differences from germline segments were determined by using the V BASE sequence directory (Tomlinson, I. M., Williams, S. C., Corbett, S. J., Cox, J. P. L. & Winter, G., MRC Centre for Protein Engineering, Cambridge, UK) and the DNAPLOT alignment package (Müller, W. & Althaus, H.-H., Köln University)

clone code*	**V_H _family**	**V_H _Segment**	**Differences from germline**	V_H_CDR3	**V_L _family**	**V_L _Segment**	**Differences from germline**	V_L_CDR3
PfNPNAl	VH3	DP46	10	DRDSSSYFDS	VkI	L12a	15	QQYNSYSGLT
NP04, NP12, NP13	VH3	DP46	10	DRDSSSYFDS	VkI	L12a		QQYNSYSGLT
R01	VH1	4M28^†‡^	28(+6)* ^§^-	DSESVAQWRY	VkIV	DPK24	43	QQSLSPVWT
R02	VH3	COS-3^‡^	27 (+3)_	GVNWCSDY	VkI	DPK9	10	QQSYSTSWT
R03	VH5	DP73	35	LYTSIYYFDS	VkIV	DPK24	7	QQYYSTPLT
R04	VH3	DP46	8	DRVTNFWSGYFDY	VkIII	DPK22	13	QQYGSSPGFT
R05	VH3	DP58	23	DSTVKTVTKMRYGLD V	VkIII	DPK22	8	QQYGSSPFT
R06	VH1	4M28^†^	12	DNYGDPGGGFDI	VkIII	DPK22	11	QQYGNSPRT
R07	VH5	DP73	9	RFWFGELYDAFDI	VkIV	DPK24	16	HQYYSTPQT
R08	VH5	DP73	34	LYTSIYYFDS	VkIII	DPK22	14	QQYGRSPWT
R09	VH3	V3-21^†^	34	DQGGGWSSEVDS	VkIII	Vg	5	QQRSNWPLT
R10	VH1	DP7^‡^	21 (+9)**	ALYGHDAFDI	VkI	DPK4	12	PKYNSALHT
NP02	VH3	DP47	36	ERPYDAFDS	VkIII	DPK22	23	QQYSTSPPMYN
NP03	VH5	DP73	40	LYTSIYYFDS	VkIII	Vg	17	KQRSKWPPIT
NP05	VH3	V3-48	14	EPRGAGTTLYFDY	VkIII	DPK22	22	QQYGGSPGYN
NP08	VH4	4.30^†^	18	DRGVSSGWTFDC	VkII	DPK16	32	MQLTAFPWT
NP09	VH4	DP71	17	FRGGVAAGYDY	VkIII	DPK22	24	QHYRESCS
NP10	VH4	DP78	29	DRVRVPYYYIDV	VkIII	DPK22	15	QQYGTSPYS
NP11	VH3	VH3-8^†^	12	DTTVTHYFDY	VkI	DPK9	21	QQSFSSPRT
NP14	VH1	DP88	20	GPGATIHYYYMDV	VkI	DPK8	18	QQLDNYPLT
NP15	VH5	DP73	36	LYTSIYYFDS	VkIII	DPK22	28	QQYGNSPPT

*Phage clones were either selected from the library at random (prefix R) or after four rounds of panning against (NPNA)_3_C-BSA, eluting with free (NPNA)_3 _peptide (prefix NP). ^† ^The segment given the best DNAPLOT match, although the segment sequence has not been verified by duplication. ^‡ ^Aligned after removal of the unusual sequence additions (see^§,^-, ^‡,^-, **). ^§ ^Figure in brackets indicates an unusual sequence addition. - Sequence has two additional codons in CDR1. - Sequence has one additional codon in CDR1. ** Sequence has three additional codons in CDR2.

### Expression and evaluation of the recombinant antibody fragment

The PfNPNA-1 sequence was transferred to the expression vector pAbHIS (as outlined in Figure 2. Purification of the V_H_/κ polypeptide was carried out on Ni-NTA agarose beads, yielding 0.5 mg of the 38 kDa V_H_/κ polypeptide/L bacterial culture.

### Fine specificity and affinity determination

**Figure 3:**
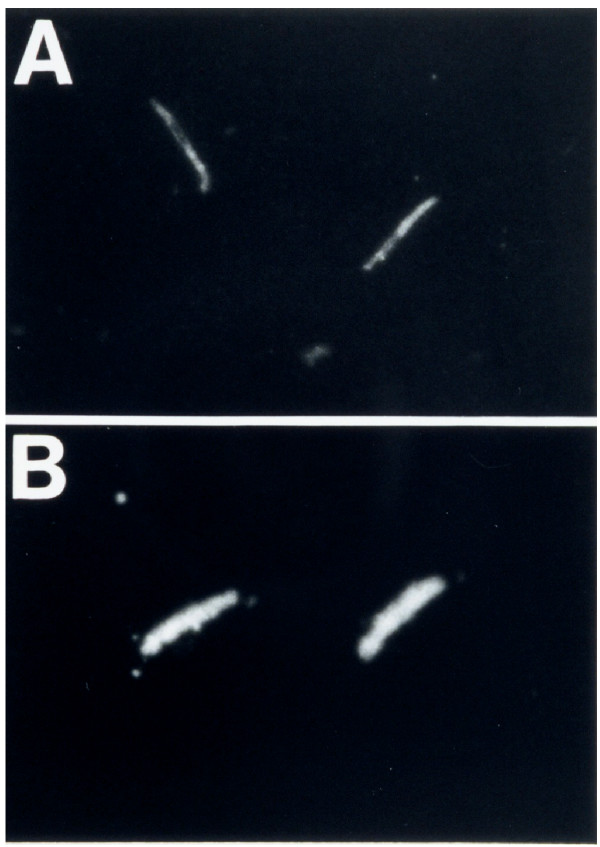
**Indirect immunofluorescence assay (IFA) on *Plasmodium falciparum *sporozoites. **Panel (A) PfNPNA-1 V_H_/κ, (B) 2A10 MAb.

Anti-sporozoite activity of the PfNPNA-1 V_H_/κ molecule was clearly evident in an immunofluorescence assay (IFA) with *P. falciparum *sporozoites (Figure 3). The human single chain monoavalent antibody (panel A) was compared with a known *in vitro *protective whole murine antibody 2A10 (panel B). The murine antibody and the recombinant PfNPNA-1 V_H_/κ molecule both labelled the parasites.

**Figure 4:**
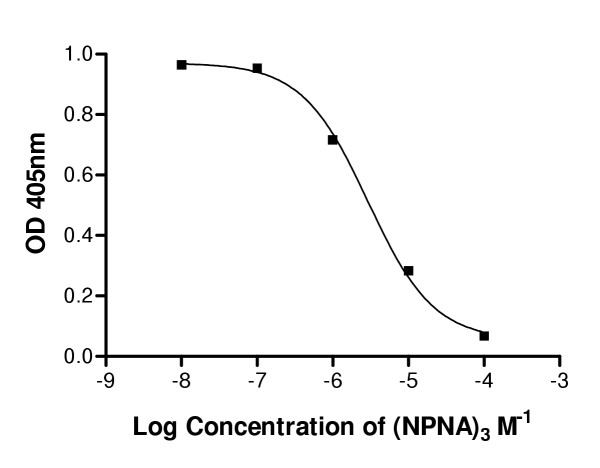
Competition ELISA.

Competitive ELISA was carried out and the IC_50 _value used to approximate the affinity of binding. Binding affinity of the monovalent PfNPNA-1 for (NPNA)_3 _compared favourably with values previously reported for a panel of conventional murine monoclonal antibodies directed against the repeat epitope [18], which also have affinities in the μM range (Figure 4).

**Figure 5:**
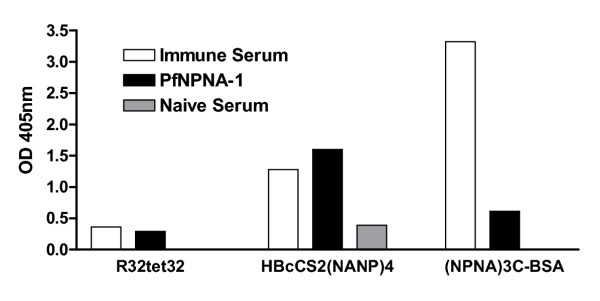
**Determination of specificity of PfNPNA-1. **The binding of immune serum (WR5), non-immune serum and PfNPNA-1 V_H_/κ to R32tet32, recombinant hepatitis core containing (NANP)_4 _peptide sequence and (NPNA)3C-BSA conjugate coated microtiter plate well was determined by ELISA essentially as described in Figure 4. The serum(s) and the recombinant PfNPNA-1 V_H_/κ were diluted 1/16 and 1/10 respectively.

Analysis of the fine specificity of the antibody PfNPNA-1 revealed weak binding to the repeat based [NVDP(NANP)_15_]_2_, R32tet32 [19], whilst binding to the (NANP)_4 _epitope contained within the hepatitis B virus nucleocapsid (C75CS2) [20] was strong. This activity profile pattern was mirrored in the protected donor serum (Figure 5). The very high binding observed with WR5 immune serum with the (NPNA)_3_C-BSA conjugate is probably due to the multivalent array of the capture ligand (i.e. multiple peptides coupled per BSA molecule), favouring more efficient retention of the antibody.

## Discussion

The recombinant antibody library construction differed from conventional antibody phage display library assembly [10-13], a pre-selection step was introduced to remove antibody inserts that were either; prematurely terminated, intact but did not translate well or were intact, translated well but failed to translocate into the bacterial periplasmic space, a prerequisite for functional display. Previously an approach towards developing a vector to select for fully intact functional sequences for antibody or peptide display had shown promise with model sequences [21], but had not been applied for large-scale random antibody library assembly. A "clean-up" vector, plasmid open reading frames expression secretion (pORFES) [14] was developed and used to remove these non-functional sequences. Up to 50% of the clones from the initial transformed library were non-functional. Some of the non-functional antibody fragments could in part be due to errors introduced during PCR amplification resulting in frame shifts. However it may be that some sequences either did not express well or did not translocate into the periplasmic space. Irrespective of the explanation, the size of the functional library was half of the total transformation events. An initial enhancement of the initial library by removing most non-functional inserts may at first appear to be a minor improvement. However, in conventional phage display the initial expansion of the library prior to panning results in a preferential growth of phage that do not make and display encoded inserts, moreover phage that lack an insert have a greater growth advantage. This results in a phage population that is greatly biased towards non-productive elements, which impacts directly on the panning efficiency. Incorporation of the pORFES step assured that only the functional (1.3 × l0^6^) sequences were subsequently transferred to the phage display vector. Panning with a functionally enhanced library resulted in very efficient enrichment and recovery. Previously it had been demonstrated that manipulating the conditions of phage production results in modulation of the density of antibody display on phage [15]. The phage library was expanded using parameters that would result in either monovalent display (0-1 antibody/phage) or multivalent display (0–5 antibodies/phage) [15] prior to initiating panning. It was anticipated that a range of antibodies with varying affinities would be present in the library, and modulating antibody display on phage would permit capture antibodies with a range of affinities and sequence diversity.

Induction of protective immunity against sporozoite challenge by exposure to radiation attenuated malaria sporozoite has been demonstrated in humans [4,7,22]. Protection is thought by most investigators to be primarily cellular in nature [23], but there is no question that antibodies with significant sporozoite neutralizing activity are elicited [22] and may play a role in protection. The antibody response is primarily directed against the repeat region of the PfCSP. Studies of subunit vaccines which induce antibodies only against the repeat region demonstrate that protective immunity can be induced in some individuals [24,25]. At the onset of this study it was proposed that the dissection of the anti-*P*. *falciparum *sporozoite antibody response by combinatorial antibody library phage display would permit individual selected antibodies to be evaluated for protective potential and the information generated could be used in vaccine design. In particular, attention was focused on antibodies against the structural motif (NPNA)_n_. Despite using two different strategies for the elution of repeat region peptide specific antibodies (acid and peptide specific) it would appear that the anti-structural repeat response by this protected individual is restricted to a single V_H_/V_L _combination observed in the panel of selected phage (n = 25). Sequencing of randomly picked phage prior to panning revealed that a diverse range of V_H _and V_L _families were represented in the library as shown in Table 2. Moreover the PfNPNA-1 V_H_/V_L _was not represented in the sampling and was only detected after enrichment.

Comparison of the monovalent PfNPNA-1 molecule with the conventional bivalent murine mAb, such as the in vitro inhibitory 2A10 against *P. falciparum *sporozoites indicates that they recognize the repeat epitope(s) with equivalent affinities [18]. The sequence revealed extensive somatic hyper mutations in both the V_H _and V_L _genes suggesting antigen driven affinity maturation. Based on these observations, PfNPNA-1 may be a good candidate to develop and evaluate as a protective antibody.

Analysis of field samples in rural Gambia [26], Thailand [27] Indonesia [28] and Kenya [29], suggest that anti-sporozoite antibody is poorly developed under natural conditions of exposure and does not protect against clinical malaria. In contrast to exposure to *P. falciparum *sporozoites under natural conditions in the field, immunization with irradiated *P. falciparum *sporozoites induces in general higher levels of antibodies against the PfCSP repeats, and does induce sterile protective immunity [4,7,30-38]. In the study by Egan et al., 3 of the 4 volunteers were protected against challenge with *P. falciparum *sporozoites. The generally accepted explanation for the lack of protection in the one volunteer is that the volunteer did not receive an adequate immunizing dose of irradiated sporozoites (less than 1000 infective bites [4,7]). However, it is of interest that this non-protected volunteer (WR1, [22])had significantly lower levels of antibodies against the PfCSP repeat than did the protected volunteer who donated cells for this study (WR5, [22]) (2.4 μg/ml vs 50 μg/ml of specific antibody). This raises the question as to whether the antibodies are markers for adequate immunization or are actually major mediators of protection. Regardless, this anti-repeat response in this protected individual appeared to be restricted to a single antibody. This does not preclude that antibodies directed against non-repeat epitopes on PfCSP and other sporozoite proteins [39] play a role in protection. It is not possible to conclude that the response against the structural repeat epitope is restricted to a single antibody of moderate affinity, since only a single protected donor has been used in this study. One may speculate that in concordance with the argument put forward by Saul [40] that the inability to recover high affinity antibody, may reflect that high affinity antibodies may not be required for protection. Due to the repetitive nature of the antigen one can further speculate that only limited affinity maturation is required to obtain physiologically relevant efficacy. The restricted recovery of antibodies is unlikely to be a technical limitation on the phage technology since others have generated panels of very high affinity human antibodies against a range of antigens [13]. Very few examples of different approaches of generating human antibodies from immune donors are described in the literature, in particular when attempting to make antibodies against the same antigen. Currently it is not possible to fully understand the limitations of a technology. Using an alternative technology of engrafting immune human PBL's directly into SCID mice from donors vaccinated against anthrax vaccine adsorbed, boosting with protective antigen (PA), recovering immortalizing antibody-producing cells via conventional hybridoma technology [41] resulted in a panel of very high affinity potent neutralizing antibodies against anthrax toxin. Independently, an antibody phage display library from a similar (not identical) immune donor PBL's was constructed and panned against PA [42] also resulted in a panel of high affinity anti-anthrax PA antibodies. This would suggest that the methodology is not limiting. However in this example, unlike CSP, the PA antigen does not contain repeating epitopes.

Further it is speculated that antibodies directed against the structural (NPNA)_n _repeat play a role in conferring protection against *P. falciparum *sporozoites in some of the protected volunteers and this protection may be associated with circulating levels of this specific antibody against the structural repeat.

Efforts are being directed towards producing a fully human IgG based on the PfNPNA-1 V_H _and V_L _domains for further in vitro and in vivo evaluation. The use of a human monoclonal antibody as a preventive measure against *P. falciparum *malaria, would be independent of factors which hinder active vaccination, such as adjuvant effects, the requirement to be effectively presented in a diverse range of human leukocyte class I and II molecules, and immunlogical antagonism [43,44]. In practice, the utility of monoclonal antibodies as anti-infectious agents is often negated by the presence and or the inevitable emergence of variants with altered surface epitopes (in particular with viral targets). Fortunately, there has never been a *P. falciparum *isolate that does not contain the (NPNA)_n _repeats on the PfCSP [45], and the number of tandem array of repeats on the PfCSP reduces the likelihood of variants arising which evade antibody recognition. This would suggest that an effective antibody directed against the repeats would be effective against all *P. falciparum. *If this restricted antibody response to the repeat epitope plays a role in preventing *P. falciparum *infection, PfNPNA-1 may be a useful prophylactic agent. Moreover, if PfNPNA-1 is shown to be protective in passive immunization in humans or monkeys as previously demonstrated for anti-*P. vivax *CSP murine mAb, NVS3 [46], it would provide a template that could be used in defining the precise conformation of the structural repeat required for the induction of desired antibodies that can neutralize parasites.

## Conclusions

Over the past 25 years the antibody response against the PfCSP repeat epitope has been pursued as a target for active vaccination, with encouraging results [47]. Our attempt to dissect the protective antibody response against the structural PfCSP repeat revealed that the response was restricted to a single V_H_/V_L _pairing, designated PfNPNA-1 encoded by V_H_3 and V_κ _I families (with evidence of somatic mutations). The affinity for the ligand was in the μM range, which in the context of a whole antibody may be more than sufficient for retention on a polyvalent surface such as the *P. falciparum *CSP. It is speculated that the induction and the maintenance of high circulating levels of antibodies against the structural PfCSP repeat may be more important than intrinsic high affinity for the ligand for protection against *P. falciparum *infection. The absence of high affinity anti-repeat antibodies is in concordance with the expected response against a multivalent antigen (i.e. sporozoite surface). Under physiological conditions a whole IgG antibody and a multimeric ligand result in bivalent binding. Such complexes can have avidities estimated to be approaching the product of two independent monomeric interactions. In this case, the 1 × 10^-6^M monovalent affinity of PfNPNA-1 may approach a theoretical higher avidity (1 × 10^-12 ^M) in the context of a whole antibody. This implies that further affinity maturation either in vivo or in vitro may not necessarily increase physiological effectiveness of the whole IgG antibody. Public health officials have acknowledged the urgency for development of an effective anti-*P*. *falciparum *malaria vaccine. One of the key criteria of such a putative vaccine may be the induction and maintenance of high levels of anti-(NPNA)n antibodies. The fully human PfNPNA-1 IgG could be used as a positive control in evaluating sera from immunized donors, or possibly be developed as a prophylactic agent that could be used alone or in combination with various vaccination strategies. One immediate hurdle for the development of such an antibody as a prophylactic would be the anticipated high cost of commercial manufacture in mammalian cells. However, advances in alternative antibody production technology may one day provide some more cost effective solutions [48,49].

With the availability of an antibody phage display library constructed from a protected individual immunized via bites of irradiated *P. falciparum *infected *Anopheles *mosquitoes, it should be possible to further dissect the antibody response against "other" sporozoite antigens [39].

## Authors' contributions

JAC was the postdoctoral researcher on this project. WOR and SLH co-investigators. ASK was the PI and recipient of the Department of Army award. All authors read and approved the final manuscript

## Disclaimer

The views and opinions expressed herein are those of the author and do not purport to reflect those of the U.S. Navy or the Department of Defense, Sanaria Inc or Avanir Pharmaceuticals Inc.
